# Profiling of plasma extracellular vesicles identifies proteins that strongly associate with patient’s global assessment of disease activity in rheumatoid arthritis

**DOI:** 10.3389/fmed.2023.1247778

**Published:** 2024-01-11

**Authors:** Onno J. Arntz, Rogier M. Thurlings, Esmeralda N. Blaney Davidson, Pascal W. T. C. Jansen, Michiel Vermeulen, Marije I. Koenders, Peter M. van der Kraan, Fons A. J. van de Loo

**Affiliations:** ^1^Department of Rheumatology, Radboud University Medical Center, Nijmegen, Netherlands; ^2^Department of Molecular Biology, Faculty of Science, Radboud Institute for Molecular Life Sciences, Oncode Institute, Radboud University Nijmegen, Nijmegen, Netherlands

**Keywords:** extracellular vesicles, rheumatoid arthritis, PGA-VAS, plasma, proteomics, vitality

## Abstract

**Background:**

Rheumatoid arthritis (RA) is an autoimmune disease characterized by chronic synovial inflammation and cartilage/bone damage. Intercellular messengers such as IL-1 and TNF play a crucial role in the pathophysiology of RA but have limited diagnostic and prognostic values. Therefore, we assessed whether the protein content of the recently discovered extracellular vesicles (EVs), which have gained attention in the pathogenesis of RA, correlates with disease activity parameters in RA patients.

**Methods:**

We identified and quantified proteins in plasma-derived EVs (pEVs), isolated by size exclusion chromatography from 17 RA patients by mass spectrophotometry (MS). Quantified protein levels were correlated with laboratory and clinical parameters and the patient’s own global assessment of their disease activity (PGA-VAS). In a second MS run, the pEV proteins of nine other RA patients were quantified and compared to those from nine healthy controls (HC).

**Results:**

No differences were observed in the concentration, size, and protein content of pEVs from RA patients. Proteomics revealed >95% overlapping proteins in RA-pEVs, compared to HC-pEVs (data are available via ProteomeXchange with identifier PXD046058). Remarkably, in both runs, the level of far more RA-pEV proteins correlated positively to PGA-VAS than to either clinical or laboratory parameters. Interestingly, all observed PGA-VAS positively correlated RA-pEV proteins were associated with the actin-cytoskeleton linker proteins, ezrin, and moesin.

**Conclusion:**

Our observation suggests that PGA-VAS (loss of vitality) may have a different underlying pathological mechanism in RA, possibly related to enhanced muscle actin-cytoskeleton activity. Furthermore, our study contributes to the growing awareness and evidence that pEVs contain valuable biomarkers for diseases, with added value for RA patients.

## Introduction

Rheumatoid arthritis (RA) is an autoimmune disease with a worldwide prevalence of 0.5–1% (1), where the immune system attacks the synovial joints, leading to joint inflammation, destruction of articular cartilage, and bone erosion (2–4). Validated laboratory parameters for RA diagnosis include elevated blood levels of rheumatoid factor IgM (IgM-RF) anti-citrullinated protein autoantibodies (ACPA). For assessing disease activity, C-reactive protein (CRP) levels and erythrocyte sedimentation rate (ESR) are measured. Clinical parameters for RA diagnosis and disease activity, as observed by the physicians, include the number of tender joint counts (TJC) and swollen joint counts (SJC). Additionally, RA patients themselves fill out the patient’s global assessment visual analog scale score (PGA-VAS), by marking a point on a 100-mm horizontal line, indicating their disease activity (5). This score reflects various factors such as fatigue, pain, synovial and systemic disease activity, and vitality (6). Besides synovial joint inflammation, involvement of other organs, such as the lungs, heart, and eyes, is a well-recognized manifestation of this disease (7). Muscle weakness is also common at all stages of RA disease activity and duration (8).

Although the etiology of RA is still not fully understood, the symmetry of arthritis and comorbidities (9, 10) suggests a contribution of circulating messengers to this disease. Most of the research studies have focused on cytokines as the main effectors in disease progression (11, 12). Recently, extracellular vesicles (EVs) have been postulated as important messengers between resident and inflammatory cells (13) and have been reported to be shed by both immune cells (14).

Extracellular vesicles are released from almost every cell type and are detectable in body fluids such as blood, synovial fluid, urine, and breast milk (15). They consist of a lipid bilayer, which protects their protein and ribonucleic acid cargo from degradation, making them ideal messengers (16). The cargo of EVs can be modulated, for example, by making changes in the intra-and extracellular microenvironment, and this makes them interesting to study as markers of joint pathology (17).

In this study, we evaluated whether plasma EV protein profiling correlates with standard laboratory and clinical parameters to reveal whether plasma EVs can provide additional diagnostic value as a diagnostic biomarker for disease activity in RA.

## Materials and methods

### Blood donors

Twenty-six RA patients fulfilling ACR/EULAR classification criteria (3) not receiving any biological treatment were recruited at the outpatient clinic of our department. Disease activity, tender joint counts (TJC), and swollen joint counts (SJC) were assessed, and DAS28 (ESR) and DAS28 (CRP) were determined by physicians. The patient global assessment visual analog scale (PGA-VAS) was determined as a patient-reported outcome. For systemic signs of inflammation, erythrocyte sedimentation rate (ESR) and C-reactive protein (CRP) levels were determined by standard laboratory blood tests. Oral informed consent was obtained after a verbal explanation of the nature and scope of the research project, according to the protocol and applicable Dutch laws. Documentation of consent in the electronic patient records to the participation in the specific protocol was considered sufficient and approved by the Institutional Review Board (IRB) of the Radboudumc (CMO region Arnhem-Nijmegen; dossier 2015–1847) and conducted according to the Declaration of Helsinki. After informed consent, an extra blood sample was taken from these patients undergoing vena puncture for clinical diagnosis/monitoring. The study material was directly pseudonymized after collection. As a control group, blood from nine sex- and age-matched healthy donors was obtained from the blood transfusion organization (Sanquin, Nijmegen, The Netherlands). An overview of the disease status and clinical parameters of the included RA patients is shown in Table 1.

**Table 1:** Demographics and clinical scores of RA patients.

	Age	Gender	Duration of disease [years]	RF	Anti-CCP	ESR	CRP	TJC	SJC	DAS28 (ESR)	DAS28 (CRP)	PGA-VAS	MS RUN
RA-1^*^	67	m	31	pos	neg	27	15	8	7	5.75	5.4	80	1
RA-2	72	m	<1	neg	neg	5	4	0	1	1.41	1.82	0	1
RA-3	53	m	<1	pos	pos	15	3	0	0	1.9	1.46	0	1
RA-4^*^	67	m	9	neg	neg	2	0	8	0	2.63	3.1	40	1
RA-5^*^	27	f	8	pos	pos	55	22	2	2	4.83	4.12	60	1
RA-6	47	f	2	pos	pos	8	9	5	1	4.12	4.44	80	1
RA-7^*^	66	f	12	pos	pos	37	10	2	2	4.42	3.71	50	1
RA-8^*^	77	f	20	pos	pos	26	4	1	5	3.75	3.01	20	1
RA-9^*^	35	f	10	neg	neg	2	1	17	6	4.73	5.45	89	1
RA-10	70	f	2	pos	pos	34	9	3	3	4.9	4.22	70	1
RA-11	84^*^	f	20	pos	pos	32	1	0	0	2.43	1.21	0	1
RA-12	55^*^	f	6	pos	pos	5	1	8	4	4.12	4.2	60	1
RA-13	78^*^	f	9	pos	pos	5	1	8	4	4.12	4.2	60	1
RA-14	79	f	<1	neg	neg	29	4	0	0	2.64	1.82	20	1
RA-15	72	f	2	pos	pos	17	3	1	0	3.24	2.72	50	1
RA-16	67	f	<1	pos	pos	65	17	0	2	4.6	3.68	90	1
RA-17	62^*^	f	5	neg	pos	18	5	3	1	3.98	3.56	50	1
RA-18	45	m	<1	pos	pos	27	53	5	4	5.38	5.47	90	2
RA-19	50	m	<1	neg	neg	2	2	2	2	2.86	3.73	85	2
RA-20	65^*^	m	29	pos	pos	40	20	3	1	4.88	4.36	75	2
RA-21	46^*^	f	5	neg	neg	42	1	8	1	5.56	4.16	77	2
RA-22	71^*^	f	7	pos	pos	10	8	5	2	4.24	4.38	70	2
RA-23	70^*^	f	6	neg	pos	38	10	0	1	3.11	2.28	20	2
RA-24	63^*^	f	21	pos	pos	16	12	1	1	3.2	3.14	30	2
RA-25	39^*^	f	5	pos	neg	35	16	3	2	4.97	4.47	80	2
RA-26	62^*^	f	8	neg	pos	24	5	4	1	4.32	3.71	50	2

^*^RA patient received methotrexate.

### Blood sample preparation

Blood was collected in ethylenediaminetetraacetic acid tubes (BD, Plymouth, United Kingdom) and within 1 h after collection centrifuged at 1,690 *g* for 10 min at 4°C to obtain plasma. Next, the supernatant (plasma) was centrifuged at 10,000 *g* for 30 min at 4°C to obtain platelet-free plasma (pfp). The obtained pfp was passed through a 0.22-μm filter (Whatman, GE Healthcare, Buckinghamshire, United Kingdom), aliquoted, and stored at −80°C.

### Plasma EV isolation

Plasma EVs were isolated by size exclusion chromatography (SEC) following our previously described protocol to ensure that the isolated pEVs were free of circulating immune complexes and cholesterol-rich lipoprotein particles (18, 19). In short, a sterile serological 10-mL pipet was stacked with 10 mL of Sepharose CL-2B (Pharmacia, Uppsala, Sweden). After washing the column with PBS containing 0.32% citrate (pH 7.4, autoclaved), 500 μL of pfp was loaded and eluted using PBS/0.32% citrate buffer; 1 mL eluate fractions were collected, and fraction 5 containing the EVs was stored at 4°C for further use.

### Protein measurement

The amount of protein in the pEV samples was measured using a Micro BCA Protein Assay Kit following the manufacturer’s protocol (ThermoScientific, Rockford, United States). pEV samples were diluted 10, 20, 40, and 80 times in 0.9% NaCl, and after 2-h incubation at 37°C, absorbance was measured using the BioRad iMark microplate reader. Protein concentration was calculated using a known standard curve of bovine serum albumin (BSA).

### Nanoparticle tracking analysis

Vesicle size distribution was estimated by the Brownian motion of particles using a NanoSight NS300 (Sysmex, Etten-Leur, The Netherlands) with Nanoparticle Tracking Analysis 3.2 software (NanoSight, Amesbury, United Kingdom). Vesicles were diluted in PBS until an optimal concentration for reliable analysis was reached (20–80 particles per frame). Each sample was measured for 60 s, using the following software settings: flow rate 50, camera level 10, and detection threshold 5.

### Western blot

To detect exosomal markers (Alix, CD9, and HSP-70), pEVs were lysed and proteins extracted in RIPA buffer (50 mM Tris–HCl, pH 7.5, 150 mM NaCl, 1% v/v Nonidet P-40, 0.5% v/v sodium deoxycholate, and 0.1% SDS) supplemented with protease inhibitor cocktail (Sigma-Aldrich, St. Louis, MO, United States). After heating for 5 min at 95°C, pEV lysates were centrifuged for 20 min at 12,000 *g*, and supernatant was collected. By 12% sodium dodecyl sulfate-polyacrylamide gel electrophoresis (SDS-PAGE), proteins were separated by electrophoresis and thereafter transferred to a 0.1-mm nitrocellulose blotting membrane (GE Healthcare, Buckinghamshire, United Kingdom). After blocking with 2% BSA in TBS-T (20 mM Tris–HCl (pH 7.4) and 0.01% Tween-20), blots were incubated with primary antibody overnight at 4°C with dilutions as mentioned in Supplementary Figure S-1. Blots were washed three times with TBS-T and subsequently incubated with HRP-tagged secondary antibody (goat-anti-mouseIgG1-HRP, Santa Cruz, 1:5,000) for 2 h at RT. Chemiluminescence reactions were carried out using the ECL™ Prime Western Blot Detection Reagent (GE Healthcare, Buckinghamshire, United Kingdom), and protein bands were visualized on a GE ImageQuant LAS-4000.

### Density gradient

For characterization, the density of isolated pEVs was determined using Optiprep Density Gradient Medium (Sigma-Aldrich, St. Louis, MO, United States) as previously described (20). In short, 5, 10, and 20% iodixanol were prepared by mixing different volumes of iodixanol with PBS. A discontinuous gradient was created by first placing 3 mL of the 5% iodixanol solution at the bottom of the tube. Subsequently, 3 mL of the 10% iodixanol was underlaid, followed by 3 mL of the 20% iodixanol. Finally, 3 mL of the 40% iodixanol solution containing EVs was placed at the bottom of the discontinuous gradient. After centrifugation in an SW 40 Ti rotor at 100,000 *g* for 18 h, 12 individual 1 mL fractions were gently collected from the top of the tubes. Each fraction was washed with PBS and centrifuged at 100,000 *g* for 90 min. Pellets were dissolved in 200 μL PBS, and EV concentration was determined by nanoparticle tracking analysis (NTA; NanoSight Ltd., Amesbury, United Kingdom).

### Proteomics

The protein content of pEVs isolated from blood donors was determined by two mass spectrometry (MS) runs (Run1: RA1-RA17 and Run2: HC1-HC9 and RA18-RA26). In short, proteins of pEVs isolated from 500 μL pfp of each donor were concentrated by acetone precipitation. Thereafter, proteins were denatured by adding 8 M urea, 0.1 M Tris pH 8.5, and DTT (10 mM). Samples were prepared using the FASP protocol (21) on a spin filter, and proteins were digested using trypsin. Resulting peptides were analyzed during a 60-min gradient of buffer B (80% acetonitrile and 0.1% formic acid) on an EASY-nLC 1000 (Thermo Fisher Scientific) coupled online to an Orbitrap Exploris 480 (Thermo Fisher Scientific), running in Top20 mode with an exclusion duration of 45 s. RAW MS data were used to query the Uniprot_HomoSapiens_154578_20160822.fasta (154,578 total entries, downloaded 22/08/16) using the free MaxQuant software (22). Label-free quantification (LFQ), the match between runs, and iBAQ quantification were enabled, and levels of each protein from individual donors were analyzed using Perseus (22). The mass spectrometry proteomics data have been deposited to the ProteomeXchange Consortium via the PRIDE (23) partner repository with the dataset identifier PXD046058. Note that the dataset was first filtered on proteins that are present minimally in four donors. Furthermore, each protein must be at least detected by four peptides.

### Bioinformatic analysis

Proteomics data were further analyzed using the online protein–protein interaction tool STRING, version 11.5.^1^ Proteomics data were further analyzed using the online protein–protein interaction tool STRING, version 11.5.1, with the following settings: minimum required interaction score of high confidence (0.700), and inclusion of proteins with more than 10 interactors.

For principal component analysis (PCA), the LFQ intensities for each sample were normalized on the level of each unique protein identity (ID) based on the average LFQ in each run over all samples for that protein ID (in other words, LFQ intensities were made relative). The PCA was made using GraphPad Prism 9.0.0 (GraphPad Software, La Jolla, CA, United States), uncorrected for sex and age or other parameters.

### Statistical analysis

All data are expressed as mean ± standard deviation (SD). Correlations of RA-pEV protein levels to clinical parameters were represented by Spearman’s rank correlation coefficient (ρ) and their *p* values. Statistical differences between RA vs. HC donors were determined using the two-tailed Mann–Whitney *U*-test. Values of *p* < 0.05 were considered to indicate statistical significance. These statistical analyses were performed using GraphPad Prism 9.0.0 (GraphPad Software, La Jolla, CA, United States).

## Results

### Characterization of EVs from plasma isolated by SEC

**Figure 1:**
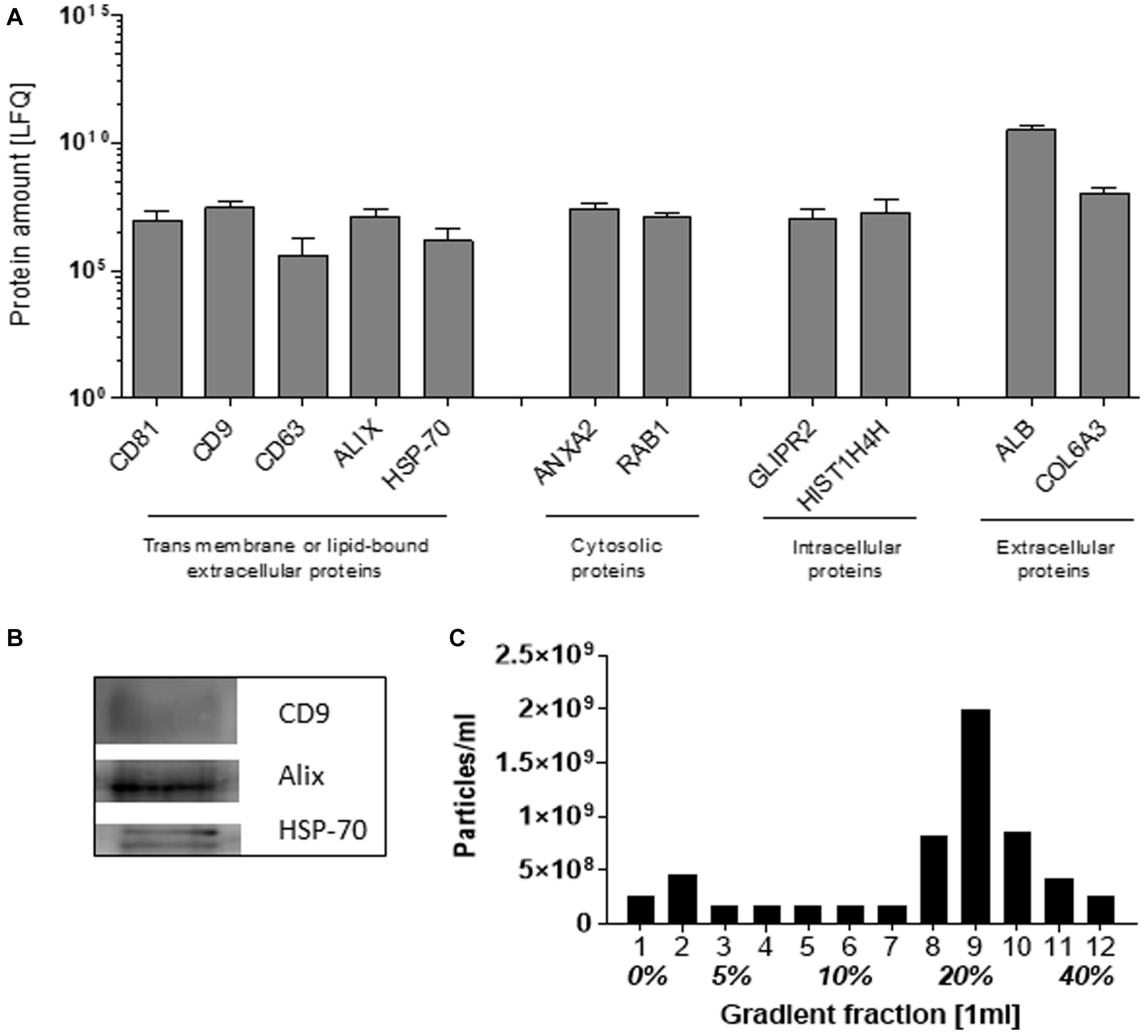
Characteristics of EVs isolated from plasma by SEC. As recommended by ISEV, at least two transmembrane or lipid-bound extracellular proteins, cytosolic, intracellular, and extracellular proteins were determined by mass spectrophotometry **(A)**. By western blot, the exosomal markers CD9, Alix, and HSP-70 were detected **(B)**. The pEV density was determined by the separation of pEVs using Optiprep Density Gradient, whereafter EVs were quantified by NTA **(C)**. For the characterization, an equal volume of 17 RA-pEVs (run1) was used.

The SEC-isolated particles were identified as extracellular vesicles based on the presence of transmembrane and lipid-bound extracellular proteins (CD81, CD9, CD63, Alix, and HSP-70), cytosolic- (ANXA2 and RAB1), intercellular- (GLIPR2 and HIST1H4H), and extracellular proteins (ALB and COL6A3), detected by mass spectrometry (Figure 1A). The expression of CD9, Alix, and HSP-70 was confirmed by Western blot (Figure 1B). Density gradient ultracentrifugation showed that the isolated pEVs had a floating density of 20% iodixanol, similar to those previously described for EVs (24). These results confirmed that the particles isolated from plasma were EVs and met the recommended ISEV guidelines (25) (Figure 1C).

### Protein profiling of EVs isolated from RA and HC donors and their correlation with disease parameters

**Figure 2:**
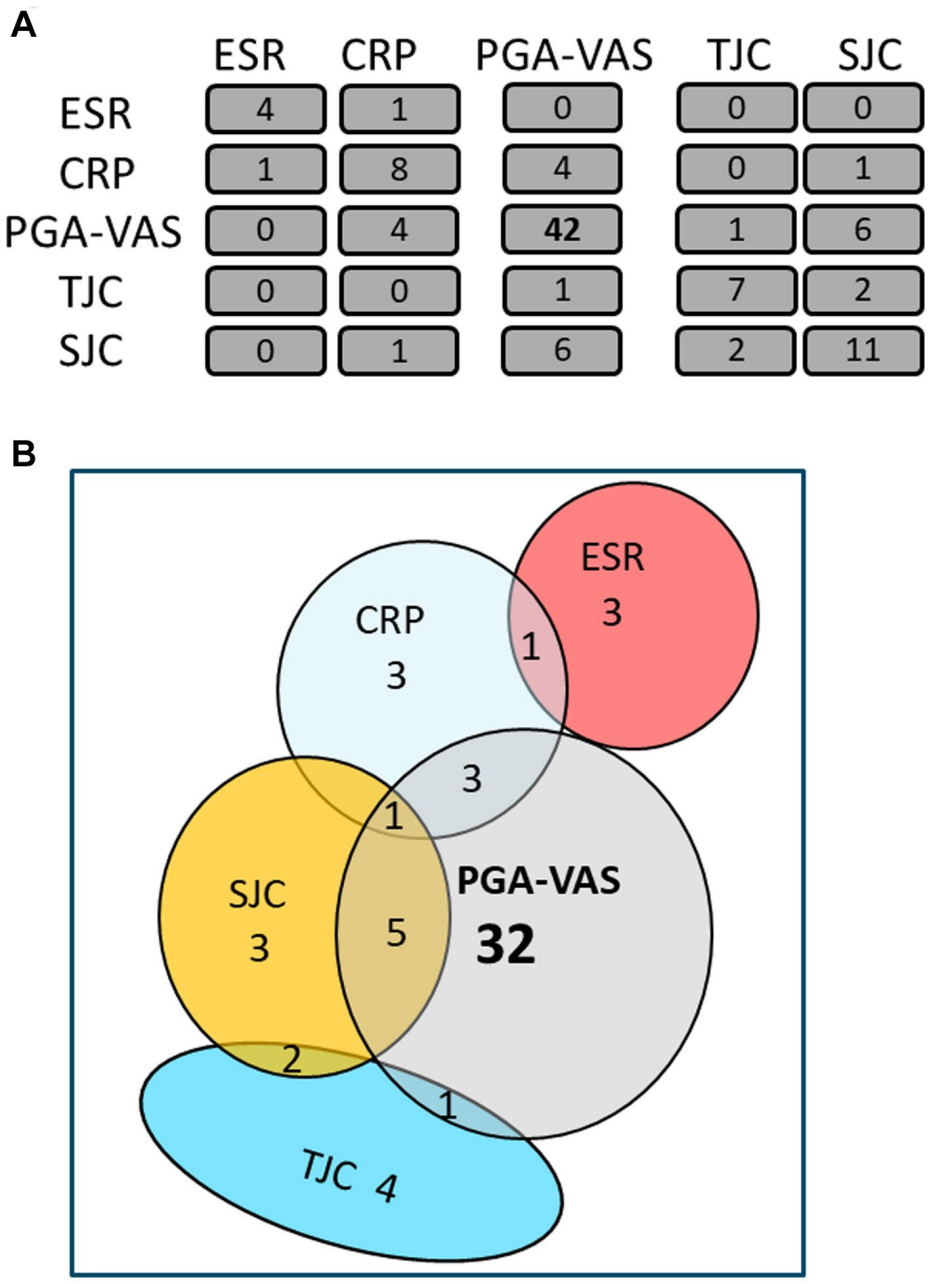
Most RA-pEV protein levels correlate positively to PGA-VAS. All RA-pEV protein levels, quantified by mass spectrophotometry, were correlated to clinical (TJC; SJC), laboratory (CRP; ESR), and own patient’s own global assessment of their disease activity (PGA-VAS; **A**). Overlap of some significantly positively correlated RA-pEV protein levels to clinical, laboratory, and patient’s own parameters was observed **(B)**.

The mean obtained particle concentrations isolated from the plasma of 17 RA patients was 1.80*10^10^ ± 1.59*10^10^ particles/mL, with a mean mode size of 114 ± 20 nm, and protein concentration of 0.41 ± 0.26 fg/particle. To investigate a possible association with RA disease activity markers, Spearman’s rank correlation coefficient (ρ) of RA-pEV protein levels to clinical parameters was calculated. An overview of the number of significantly correlated RA-pEV proteins to each disease marker is shown (Figure 2A). Strikingly, 42 protein levels of RA-pEVs positively correlated to the patient-reported PGA-VAS, while a relatively lower number of proteins correlated to the other clinical and laboratory disease activity parameters with only minimal overlap between them (Figure 2B).

**Figure 3:**
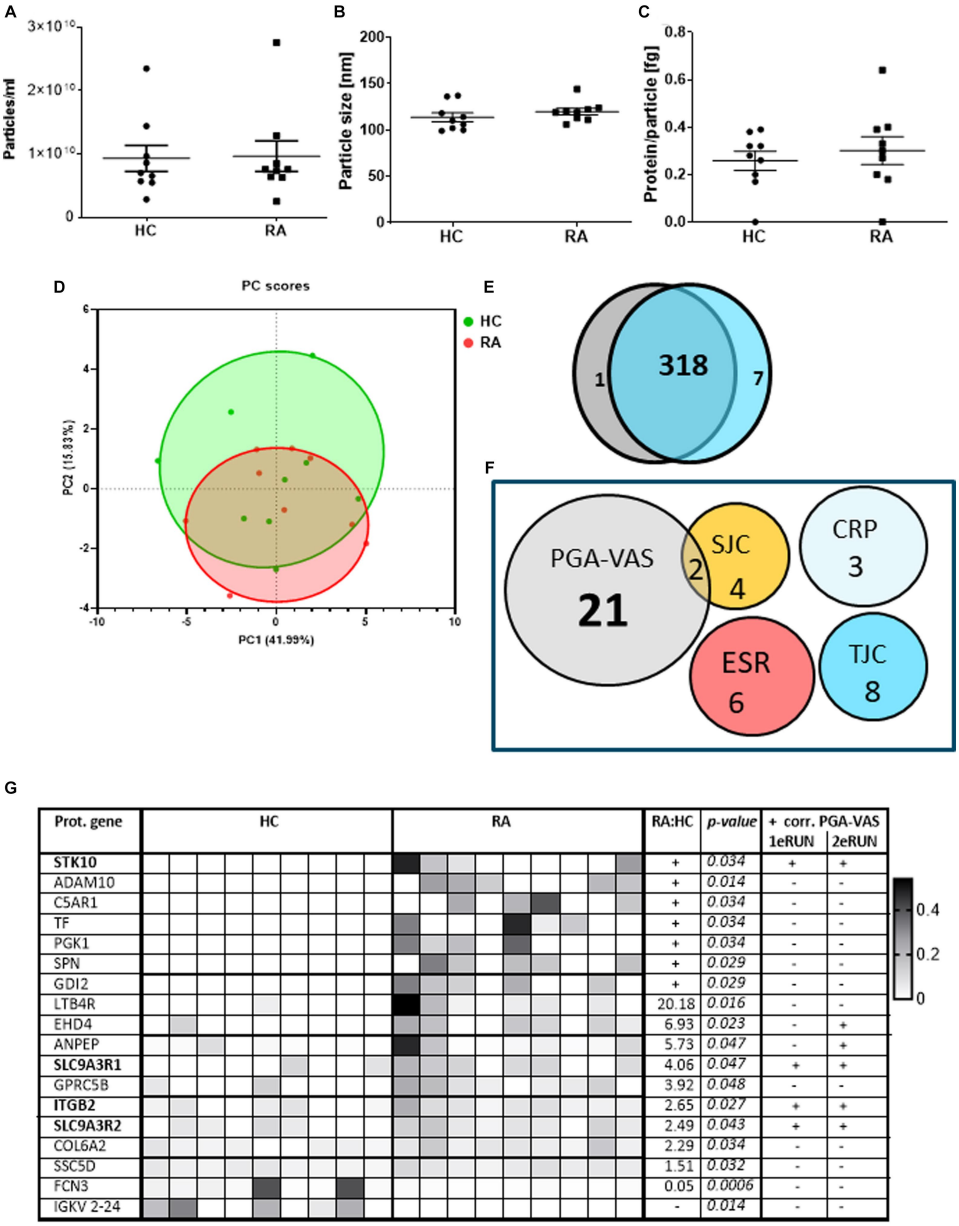
Protein profiling of EVs isolated from RA and HC donors and their correlation with disease parameters. Amount of particles **(A)**, mode particle size **(B)**, and protein content per particle **(C)** from pEVs isolated from 500 μL pfp of HC (*n* = 9) or RA (*n* = 9) were determined by NTA and micro-BCA protein assay. After preselection (each protein must be at least detected by two peptides and present in at least four donors) in a PCA plot **(D)** individual pEV donors are shown (RA in red; HC in green). Venn diagram of all pEVs proteins of HC (gray) or RA (blue) pEV donors, detected by mass spectrophotometry **(E)**. RA-pEV protein levels quantified by mass spectrophotometry were correlated to clinical (TJC; SJC), laboratory (CRP; ESR), and patient’s own global assessment of their disease activity (PGA-VAS; **F**). All significant RA-pEV proteins (vs. HC) are shown in a heatmap **(G)**. Normalized protein levels highly present are colored black and low, white. Ratio of mean RA vs. HC protein level is added including their statistical *p* values. For each RA-pEV protein, a significant correlation to PGA-VAS is added from the first and second MS runs. Statistically significant differences (*p* values) between RA and HC were determined by a two-tailed Mann–Whitney *U* test.

To confirm these findings, we re-analyzed an earlier proteomics dataset from pEVs, obtained from a previous patient cohort. This cohort included nine RA patients and also age- and sex-matched HC donors to investigate the specificity of RA-pEVs (Run2). The obtained particle concentrations isolated from the plasma of nine RA patients (9.63*10^9^ particles/mL) and nine HC (9.30*10^9^ particles/mL) by NTA were similar (Figure 3A). Additionally, the mean mode size was comparable (RA: 120 ± 14 nm; HC: 114 ± 10 nm; Figure 3B). Our modified SEC yielded highly pure pEVs whereof the protein content per particle was not different between RA- (0.30 ± 0.16 fg) and HC-pEVs (0.26 ± 0.11 fg; Figure 3C).

Principal component analysis showed that, based on the pEV protein levels, there is (i) a large variation between the individual samples and (ii) no clear distinction between samples from RA and HC-pEVs (Figure 3D), which was confirmed by the high overlapping amount of detectable proteins (318/326) detectable on RA- and HC-pEVs (Figure 3E).

By focusing on the previously observed link to PGA-VAS, similarly, most of the RA-pEV protein levels were positively correlated to this disease parameter (Figure 3F).

Of all significantly enriched pEV protein levels, as shown in a heatmap (Figure 3G), four (STK10, SLC9A3R1, ITGB2, and SLC9A3R2) were positively correlated to PGA-VAS in both MS runs (Figure 3G). Furthermore, eight proteins were uniquely present in RA- or HC-pEVs. Six pEV protein levels (STK10, ADAM10, C5AR1, TF, PGK1, SPN, and GDI2) were significantly enriched, and one (FCN3) was significantly diminished in RA.

### Biological analysis of the PGA-VAS positively correlated RA-pEV proteins

**Figure 4:**
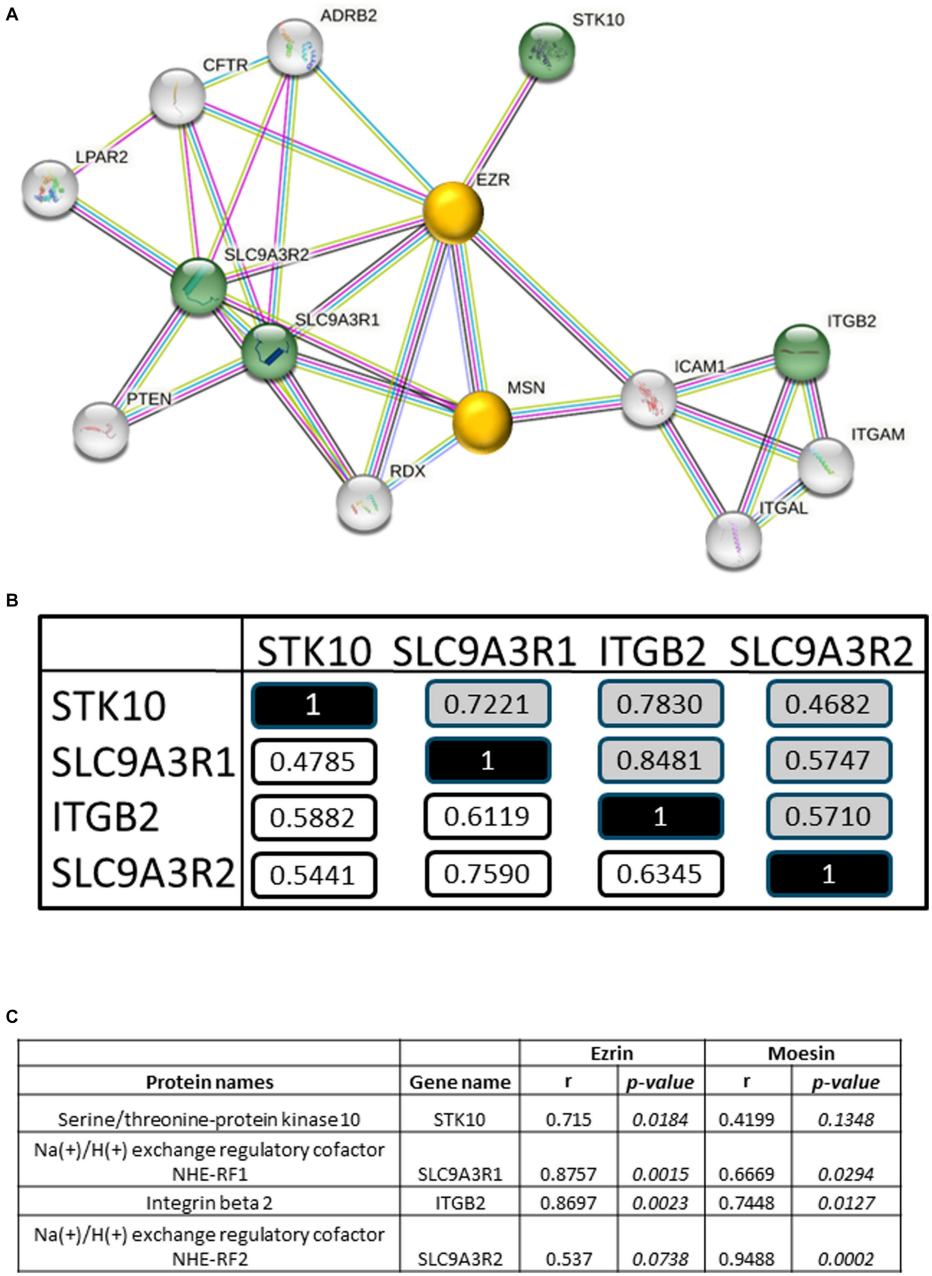
Biological analysis of enriched RA-pEV proteins. An overview of protein–protein interaction of the significantly enriched and positively correlated RA-pEVs proteins to PGA-VAS using STRING **(A)**. Green nodules are the enriched and positively correlated RA-pEVs proteins to PGA-VAS, and white nodules are interacting proteins. The central proteins are marked as yellow nodules. Intern correlation of the four RA-pEV proteins was calculated **(B)**. In white are correlations of the first run (*n* = 17) and in gray are correlations of the second run (*n* = 9). Correlation of the central proteins, ezrin and moesin, to significantly enriched RA-pEV proteins **(C)**. Correlation was calculated by Spearman’s rank correlation coefficient (ρ) and their *p* values.

Identification of interaction networks for the statistically four positively correlated RA-pEV proteins IDs to PGA-VAS showed ezrin and moesin as central proteins, using STRING analysis (string-db.org; Figure 4A). The four significantly enriched RA-pEV protein levels showed a positive correlation to each other in the first and second MS runs (Figure 4B), indicating that these proteins were simultaneously enriched in pEVs during the RA disease process. The significant correlation observed between ezrin and moesin EV levels and the enriched RA-pEV protein levels (Figure 4C) confirms their close functional association as predicted by String analysis.

## Discussion

Our study demonstrated, in two different MS runs, that a higher number of proteins on RA-pEVs positively correlated with PGA-VAS [#42; 23] than to any of the other clinical disease parameters (TJC [#7; 8], SJC [#11; 6], CRP[#8; 3], and ESR[#4; 6]). By comparison of RA-pEVs to sex- and age-matched healthy controls (HC), no differences in amount, particle size, and protein concentration were observed. Both RA and HC exhibited high individual donor variability in their overall protein profiles of pEVs. Although approximately 97% of the pEV proteins overlapped between both groups, we identified seven unique RA-pEV proteins (STK10, ADAM10, C5AR1, TF, PGK1, SPN, and GDI2) and nine significantly enriched (LTB4R, EHD4, ANPEP, SLC9AR1, GPRC5B, ITGB2, SLC9AR2, COL6A2, and SSC5D). One pEV protein was significantly diminished on RA-pEVs (FCN3), and one was undetectable, compared to HC-pEVs. The enriched RA-pEV proteins, STK10, ITGB2, and SLC9A3R1, were previously locally observed to be enhanced in synovial fluid (SF) from RA patients, while in that study, SLC9A3R2 was undetectable (Supplementary Figure S-2) (26). This observation suggests that some circulating RA-EVs might either derive from the inflamed joint (s) or vice versa diffuse into the joint during swelling. Interestingly, four significantly enriched RA-pEV proteins (STK10, ITGB2, SLC9A3R1, and SLC9A3R2) were also significantly positively correlated to the clinical parameter PGA-VAS in both MS runs. These proteins are all (in) direct linked to ezrin and moesin, which play a structural and regulatory role in the rearrangement of plasma membrane flexibility (27).

Verification of these proteins using Vesiclepedia^2^ showed that ITGB2 was previously found on circulating plasma exosomes, SLC9A3R1 was detected on plasma microparticles, and SLC9A3R2 was earlier detected on serum exosomes (Supplementary Figure S-3). It seems that we are the first who detect STK10 on circulating EVs and that some RA-pEV proteins are involved in the vitality score (PGA-VAS), which sheds light on a potential biological factor in the “subjective” measure of global disease activity in RA.

Extracellular vesicles are secreted into the circulation by various cells, and they can carry proteins serving as biomarkers for various diseases or conditions. It is less burdensome for patients and more convenient for medical professionals to use plasma, instead of synovial fluid for biomarker detection. Circulating EVs in RA patients have been previously studied. Large TLR3 fragments were observed in EVs isolated from the serum of active RA patients (28). Others have detected 278 exosomal proteins in plasma EVs where 32 proteins were significantly enriched in RA-EVs, compared to HC-pEVs (29). Unfortunately, we were unable to detect these proteins to confirm their data. The reason could be that their study did not mention exosome markers (tetraspanins), and, in our opinion, the presence of co-isolated plasma proteins cannot be excluded from their study. The conventional SEC EV isolation method can co-isolate proteins (30), resulting in the impurity of these isolated EVs resulting in inaccurate results. This was confirmed by our previous study where we observed co-isolated biologicals by SEC in the pEV-isolated samples, which are undetectable using the optimized SEC (19).

Based on freely available data,^3^ it seems that the pEV proteins ITGB2 and STK10 originally came from the NK cells (Supplementary Figure S-4). The fact that STK10, ITGB2, SLC9A3R1, and SLC9A3R2 are related to inflammation and arthritis strengthens our findings that these proteins reflect the arthritic process. STK10 may contribute to the dysregulation of apoptosis possibly involved in RA (31). ITGB2 (CD18) a receptor for ICAM1, ICAM2, ICAM3, and ICAM4, is a potential mechanistic biomarker for vasculitis (32). SLC9A3R2 is a negative regulator of endothelial proliferation (33), and SLC9A3R1 is involved in the positive regulation of NF-kB in vascular inflammation and in the IL-6 production in human smooth muscle cells (34). SLC9A3R1 and SLC9A3R2 connect plasma membrane proteins with members of the ezrin/moesin/radixin family, thereby helping to link them to the actin cytoskeleton and to regulate their surface expression. This may also explain the high degree of discordance between PGA-VAS and physician global assessment of RA (35), as, for example, muscle function is not included in the clinical assessment of RA. PGA-VAS is considered a subjective health assessment in which the patient’s judgment is crucial (36). Previous studies have shown that PGA-VAS is associated with symptoms such as fatigue, pain, anxiety, loss of vitality, and physical functions (37, 38). This makes it very intriguing that we found a link between a specific set of RA-pEV proteins with PGA-VAS, potentially pointing to an underlying mechanism (endothelium?) not measured by the standard clinical and laboratory parameters.

By protein interaction network analysis, we identified that significantly enriched and PGA-VAS positive correlated RA-pEV proteins were linked to ezrin and moesin. These proteins belong to the ERM (ezrin–radixin–moesin) family, with ezrin and moesin detectable in both RA- and HC-pEVs (1.79 times enriched on RA-pEVs but not significantly different from HC), while radixin was undetectable. We found that ITGB2 and SLC9ASR1 significantly correlated with both ezrin and moesin, whereas STK10 was correlated only with ezrin, and SLC9A3R2, only with moesin. SLC9A3R1 (also known as NHERF1 and EBP50 is a scaffolding protein of ezrin), also enriched in RA-pEVs, is known to be involved in the regulation of smooth muscle cell migration and in atherosclerosis, a disorder with pain and fatigue as comorbidity symptoms (39, 40).

The expression of dystrophin, the missing or defective protein in Duchenne muscular dystrophy, is not limited to muscle cells but also extends to vascular endothelial cells. The identification of moesin as a target for intervention in muscular dystrophy strengthens our findings, linking the PGA-VAS-related RA-pEV proteins to our data on chronic fatigue (42). Emerging evidence shows that peripheral endothelial dysfunction is associated with fatigue and hyperalgesia (43). Ezrin fits right in as it is a downstream kinase of the EGF receptor (44) and is involved in the barrier function of the vascular endothelium (45). As women with chronic stress-induced exhaustion have enhanced EGF levels (46), a fatigue link to ezrin seems plausible.

For treatment of RA, NSAIDs are the most widely used medicines, but long-term exposure contributes to muscle weakness (8, 47). Therefore, investigating the effect of RA treatment on the RA-pEV protein cargo, especially ERM-related proteins, would be interesting.

A limitation of this study is the small group of patients in which the enriched RA-pEV proteins (compared to HC) were observed. However, it is worth noting that these enriched pEV proteins are specific to RA patients and not found in patients with osteoarthritis or systemic sclerosis (not shown). Further research should prioritize the validation of these pEV proteins in a larger cohort study to determine their prognostic value for disease course and therapy response. The significantly enriched RA-pEV proteins that positively correlated with PGA-VAS were observed in two independent sets of RA patients, suggesting their potential as biomarkers for RA symptoms not captured by standard laboratory and clinical assessments.

## Conclusion

Our data indicate, for the first time, that RA-pEVs may be linked to the self-reported disease activity of RA patients. The analysis of proteins in circulating EVs can contribute to a better understanding of RA pathogenesis and their role as intercellular messengers and may yield valuable biomarkers, especially for addressing the symptom of fatigue as assessed by PGA-VAS.

## Data availability statement

The data presented in the study are deposited in the PRoteomics IDEntifications (PRIDE) Archive database (http://www.ebi.ac.uk/pridel), accession number pxd046058. The numbering of individual pEV donors is available in Supplementary material Table S-1.

## Ethics statement

The studies involving humans were approved by Institutional Review Board (IRB) of the Radboudumc (CMO region Arnhem-Nijmegen; dossier 2015–1847) and conducted according to the Declaration of Helsinki. The studies were conducted in accordance with the local legislation and institutional requirements. Written informed consent for participation was not required from the participants or the participants’ legal guardians/next of kin because written informed consent for participation was not required for this study in accordance with the national legislation and the institutional requirements.

## Author contributions

OA, EB, RT, PK, MK, and FL participated in the design of the study. OA, PJ, RT, MK, and FL contributed to the experimental methods. OA and FL performed data analysis and wrote the manuscript. OA, EB, PJ, RT, MV, PK, MK, and FL contributed to discussions and approved the manuscript. All authors contributed to the article and approved the submitted version.

## Footnotes

^1^www.string-db.org

^2^http://microvesicles.org

^3^proteinatlas.org
