# Supplementary figures and images for: Profiling of plasma extracellular vesicles identifies proteins that strongly associate with patient’s global assessment of disease activity in rheumatoid arthritis
Source: Front Med (Lausanne). 2024 Jan 11;10:1247778. doi: 10.3389/fmed.2023.1247778 (PMC10808582; doi:10.3389/fmed.2023.1247778)

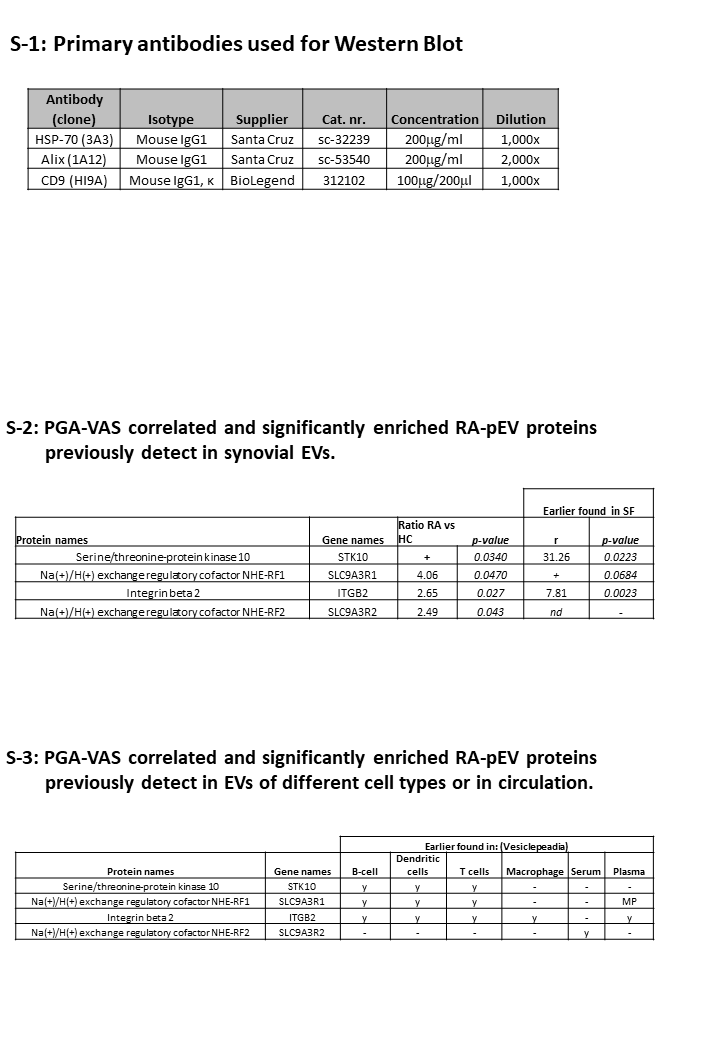

Supplement: Supplementary file 2 [file Image_1.TIF]

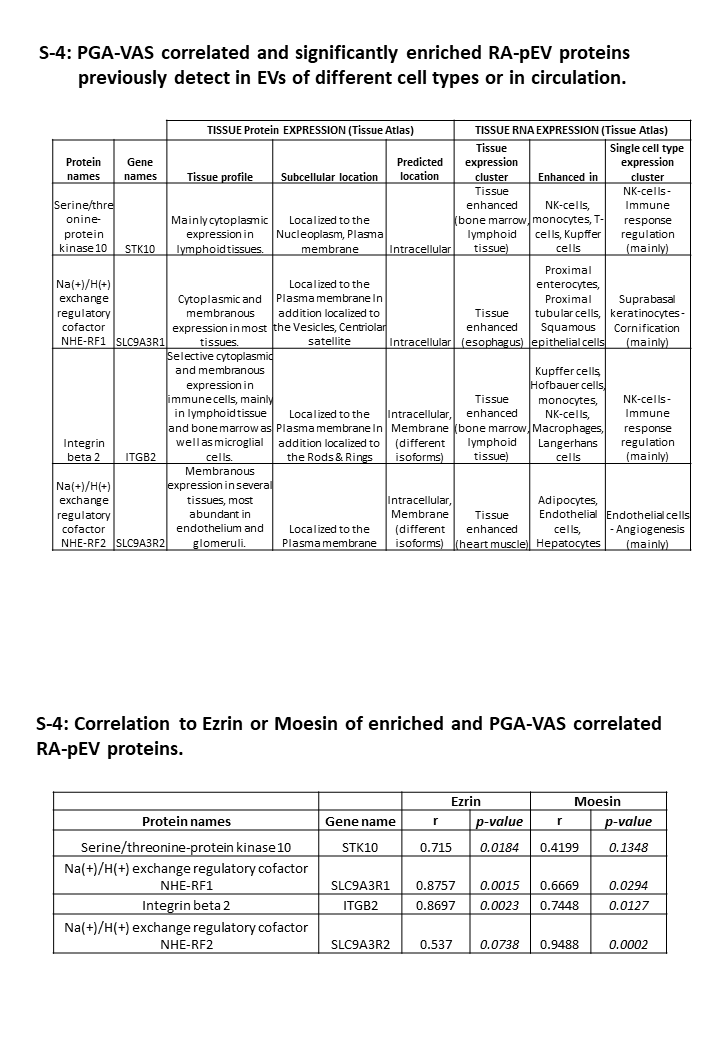

Supplement: Supplementary file 3 [file Image_2.TIF]
